# A 5-lncRNA Signature Associated with Smoking Predicts the Overall Survival of Patients with Muscle-Invasive Bladder Cancer

**DOI:** 10.1155/2021/8839747

**Published:** 2021-01-18

**Authors:** Haoyue Sheng, Guiming Zhang, Yongqiang Huang, Lijiang Sun, Guohai Shi, Dingwei Ye

**Affiliations:** ^1^Department of Oncology, Shanghai Medical College, Fudan University, China; ^2^Department of Urology, Fudan University Shanghai Cancer Center, China; ^3^Department of Biochemistry and Molecular Biology, Mayo Clinic College of Medicine and Science, USA; ^4^Department of Urology, The Affiliated Hospital of Qingdao University, China

## Abstract

Increasing evidence demonstrated that noncoding RNA is abnormally expressed in cancer tissues and serves a vital role in tumorigenesis, tumor development, and metastasis. The aim of the present study was to determine an lncRNA signature in order to predict the overall survival (OS) of patients with muscle-invasive bladder cancer (MIBC). A total of 246 patients with pathologically confirmed MIBC in The Cancer Genome Atlas (TCGA) dataset were recruited and included in the present study. We choose patients who have smoked less (including never smoking) or more than 15 years. A total of 44 differentially expressed lncRNAs were identified with a fold change larger than 1.5 and a *P* value < 0.05 through the limma package. Subsequently, a comparison between patients with no tobacco smoke exposure for <15 years and patients who had been exposed to tobacco smoke for >15 years was performed by using the matchIt package. Among the 44 differentially expressed lncRNAs, 5 lncRNAs were identified to be significantly associated with OS. Based on the characteristic risk scores of these 5 lncRNAs, patients were divided into low-risk and high-risk groups and exhibited significant differences in OS. Multivariate Cox regression analysis demonstrated that the 5-lncRNA signature was independent of age, tumor-node metastasis (TNM) staging, lymphatic node status, and adjuvant postoperative radiotherapy. In the present study, a novel 5-lncRNA signature was developed and was demonstrated to be useful in predicting the survival of patients with MIBC. If validated, this lncRNA signature may assist in the selection of a high-risk subpopulation that requires more aggressive therapeutic intervention. The risk scores involved in several associated pathways were identified using gene set enrichment analysis (GSEA). However, the clinical implications and mechanism of these 5 lncRNAs require further investigation.

## 1. Introduction

Bladder cancer is diagnosed in ~74,000 patients in the USA and in >430,000 patients worldwide, making it the 4th most common cancer in men and the 11th most common cancer in women [1]. In China, there were ~80,500 new cancer cases including 62,100 in men and 18,400 in women in 2015 [2]. The standard treatment for bladder cancer depends on the tumor (T) stage [3]. The preferred treatment for non-muscle-invasive bladder cancer (NMIBC) is transurethral resection of the bladder tumor (TURBT) combined with intravesical chemotherapy, when the T stage is <T2 [4]. Depending on the pathological grade and other risk factors, 24-49% of patients undergo repeat transurethral resection of the bladder tumor (Re-TURBT) [5]. Approximately 25% of patients are diagnosed with muscle-invasive bladder cancer (MIBC) and have a less favorable prognosis, with a 5-year survival rate < 40% [6]. Although there are improvements in surgical techniques and adjuvant therapy, the complexity and high cost of the procedure remain extremely challenging, and the treatment has not been advanced in decades [7]. Therefore, in order to further comprehend and predict patient prognosis and to develop novel biological therapies, there is an urgent requirement to identify reliable biomarkers for MIBC.

Although the etiology of bladder cancer has not been completely elucidated, bladder cancer has been linked to tobacco smoke (TS) [8], exposure to radiation or chemicals, and other risk factors, such as environmental exposure. Studies have demonstrated that TS is closely associated with the occurrence and development of bladder cancer. It is reported that TS is one of the most significant single risk factors for bladder cancer with a causal relationship of 40-60% [9]. The risk of bladder cancer among smokers has been estimated to be 5 times higher compared with that among nonsmokers [10]. A positive smoking history was identified as one of the independent risk factors for bladder tumor recurrence after transurethral resection of the bladder tumor. Furthermore, refraining from smoking for 15 years or more reduced the risk of tumor recurrence in former smokers with NMIBC regardless of the intensity or duration of smoking [11].

There have been many studies that have demonstrated that the expression of a single gene is associated with bladder cancer, and this includes not only RNA encoding but also noncoding RNA [12]; the present study has no way to clearly explain its OS mechanisms. However, owing to the complex pathological processes of bladder cancer, the use of a single gene to clarify the appearance of OS would more readily introduce a false-positive result. For MIBC, the understanding of long noncoding RNAs (lncRNAs) remains to be limited, and to the best of our knowledge, at present, there is no relevant study regarding lncRNA characteristics for the prediction of OS using a publicly available dataset. Therefore, the present study was aimed at acquiring more information from the available datasets. By using The Cancer Genomic Atlas (TCGA) dataset, the present study attempted to establish whether there was a set of lncRNAs that could distinguish between more aggressive phenotypes and poor survival outcomes in patients. Following this, an lncRNA novel signature was highlighted, which was demonstrated to accurately predict the survival of patients with MIBC.

## 2. Materials and Methods

### 2.1. Patients and Tissue Samples

The lncRNA expression information of bladder cancer (BLCA) in TCGA bladder cancer (BLCA) RNA sequencing database and the full clinical dataset of TCGA BLCA using the cut-off (August 11, 2016) are available from The Atlas of Noncoding RNAs in Cancer (TANRIC) (http://bioinformatics.mdanderson.org/main/TANRIC:Overview) [13] and the UCSC Xena website (http://xena.ucsc.edu/), respectively. The exclusion criteria were set as follows: (1) if the histological diagnosis was not MIBC (*n* = 1), (2) samples with clinical data but without T stage data (*n* = 1), and (3) missing important clinical data, including diagnosis subtype (*n* = 5 cases). Overall, the present study recruited 246 patients with lncRNA expression data and pertinent clinical information available. Furthermore, we also excluded the lncRNA expression information of the adjacent normal tissues from 23 patients from TCGA dataset.

### 2.2. Statistical and Data Mining Analyses of TCGA BLCA lncRNA Profiles

The neoplasm histological grade, pathological stage, and diagnostic subtype were used to confirm the clinicopathological features of the two groups by using the matchIt package (ratio = 1, caliper = 0.05). Finally, 13 vs. 13 patients (no relapse or metastasis with smoking more than 15 years vs. relapse or metastasis with smoking less than 15 years including no smoking) were generated from the matched clinicopathological features of the patient [11].

The present study utilized the “limma” package to identify differentially expressed lncRNA between the two previously mentioned groups [14]. The fold change and *P* values were FC > 1.5 and *P* value < 0.05. The lncRNA “Pheatmap” package was used to plot the heat map of differentially expressed lncRNAs. The different lncRNAs were analyzed in a large sample using two items of logistic regression and a single-factor Cox proportional hazards regression, using the median expression value as the cut-off point, to determine potential lncRNA associated with OS by using the “osgeneral” package. The coefficient of each lncRNA was measured with a multivariable Cox regression hazard model with all selected lncRNAs.

The risk score was calculated as the following formula [15]:

(1) $$\begin{array}{c} \text{Risk score}=\overset{n}{\underset{i}{\sum }}\left(\beta_{i}x_{i}\right), \end{array}$$

where *β*_*i*_ indicates the coefficient and *x*_*i*_ refers to the relative expression value of the corresponding gene, dividing the patients into a high-risk group or a low-risk group with the median as a cut-off point. In this formula, value *m* refers to the total number of genes in the panel, and the coefficient *n* is the regression coefficient in all selected lncRNAs in the multi-Cox regression analysis. Pearson's chi-square test was used to examine the association between clinicopathological features of patients and the 5-lncRNA signature risk score. The unpaired *t*-test was used to compare the risk score between two different subtypes of patients. The Kaplan-Meier survival was used to assess the survival distribution between the classification sets. The log-rank test was used to assess the statistical significance between stratified survival sets. The area under the receiver operating characteristic (ROC) curve (AUROC) was calculated from the ROC curve using the “survivalROC” package. *P* value < 0.05 was considered significant. All of the above data were processed by using packages in R 2.15.3 and 3.5.3 and SPSS for Windows, version 22 (IBM Corp., Armonk, NY, USA).

### 2.3. GSEA KEGG Pathway Analysis Settings

Gene set enrichment analysis (GSEA) was implemented using Java software (http://software.broadinstitute.org/gsea/index.jsp), and the gene expression file and phenotype file (high/low-risk group) were prepared according to the guideline of GSEA. The parameters were set as 1000 permutations, at least 5 genes in a single pathway, and used the KEGG pathway.

## 3. Results

### 3.1. Patient Characteristics

All of the 246 patients included in the present study had pathologically confirmed MIBC. The mean age at the time of diagnosis was 68.04 years (SD, 10.945), and the mean follow-up time was 21.18 months (Table 1). In addition, a total of 23 patients with adjacent nontumor tissue were not involved in the screening for differential expression of lncRNAs among bladder cancer tissue. During a follow-up, 104 patients died.

### 3.2. Differentially Expressed lncRNAs of MIBC between Patients with/without Relapse- or Metastasis-Related TS

The expression of lncRNAs in 13 vs. 13 MIBC tissues (with vs. without relapse- or metastasis-associated TS) was detected in the present study (Table S1). A total of 44 differentially expressed lncRNAs were screened, and the differential values were set at over log(FC) = log2(0.5) and *P* < 0.05. Of the 44 lncRNAs, 11 lncRNAs (25%) were downregulated and the remaining 33 lncRNAs (75%) were upregulated (Table 2).

### 3.3. Establishment of lncRNA Signatures Associated with Survival in Patients with MIBC

**Figure 1:**
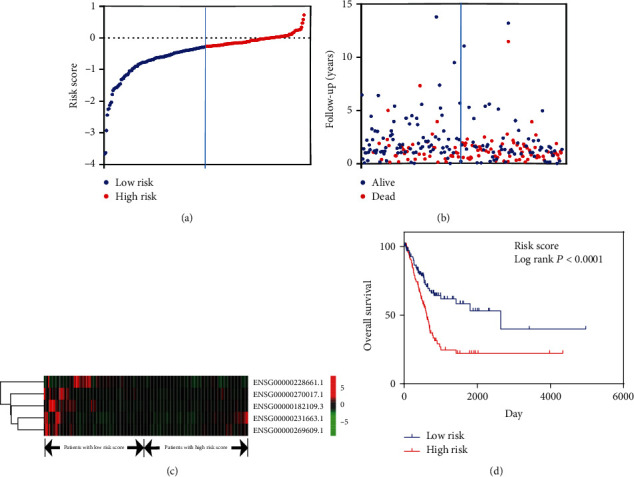
The distribution of the risk score in the signature and its relationship with overall survival.

In order to determine candidate lncRNAs with prognostic features, differentially expressed lncRNAs were further analyzed using binomial logistic regression and univariate Cox proportional hazards regression. A total of five lncRNAs were identified to be significantly associated with OS (*P* < 0.05). Of these five lncRNAs, one (AC090587.5) was positively correlated with OS and the remaining four lncRNAs (RP5-827C21.4, AL365277.1, RPARP-AS1, and CTD-2576F9.2) were negatively correlated with OS. According to the risk score, the median risk was taken as the critical value, and the patients were divided into a high-risk group (*n* = 123) and a low-risk group (*n* = 123). Compared with the low-risk group, the high-risk group of patients exhibited a poorer OS (Figure 1). There were significant differences in the distribution of age, lymph node status, and American Joint Committee on Cancer (AJCC) T stage, but not in the pathological stage (Table 3).

In addition, univariate and multivariate Cox regression analyses were performed to examine the prognostic impact of the 5-lncRNA signature on OS. As summarized in Table 4, univariate analysis demonstrated that the 5-lncRNA signature, age, tumor subtype, AJCC T stage, lymphatic status, lymphovascular invasion, and pathological stage were significantly associated with patient OS; however, sex, histological grade, extracapsular extension, family history, and smoking status were not. In a subsequent multivariate analysis, the 5-lncRNA signature continued to indicate significance by a two-sided log-rank test (hazard ratio (HR), 2.210; 95% confidence interval (CI), 0.845-2.430), revealing that the 5-lncRNA signature was an independent prognostic factor.

**Figure 2:**
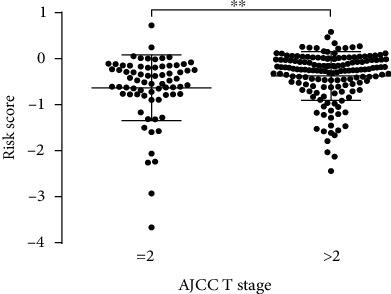
The relationship between the risk score and T stage.

**Figure 3:**
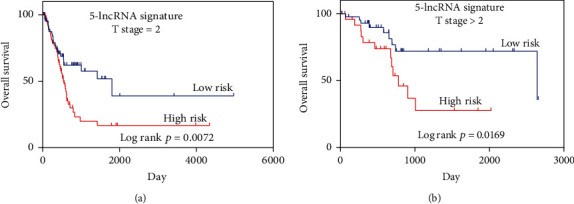
The risk score can also predict OS under different T stages.

Although by multivariate analysis, this 5-lncRNA-based risk score was not associated with the AJCC T stage, a positive correlation was found for this signature when patients were stratified according to the T stage (T ≤ 2 vs. T > 2; Figure 2, *P* = 0.0067, unpaired *t*-test). Patients with a high T stage (T > 2) exhibited higher risk scores. Therefore, a subgroup analysis was performed to determine whether the prognostic value of the 5-lncRNA signature is suitable for all patients, irrespective of the AJCC T stage. As illustrated in Figure 3, it was identified that patients with T stage < 2 were statistically significant (*P* = 0.0072 vs. *P* = 0.0169) from patients with T stage ≥ 2.

In addition, its validity in the prediction of relapse-free survival (RFS) was examined between the low- and high-risk score groups (Figure S1). A clear statistical difference between the two groups (*P* < 0.001) was identified. Additionally, in a multivariate Cox regression analysis, the 5-lncRNA signature remained a prognostic factor for RFS independent of the AJCC T stage, lymphatic vessel status, pathological stage, and lymphovascular invasion (Table 5).

**Figure 4:**
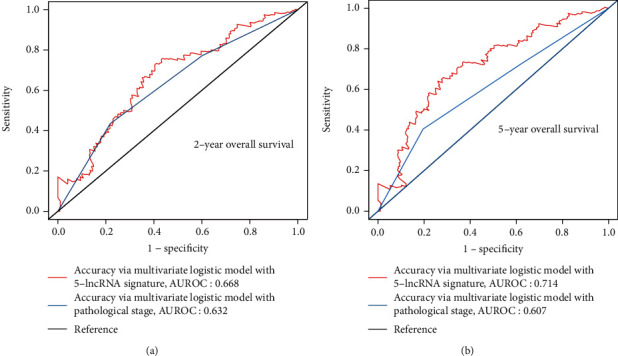
Risk score model is more efficient than TNM staging in predicting OS.

Finally, the ROC package was implemented in order to compare the accuracy of the predictions calculated by the multivariate logistic model with the 5-lncRNA signature or with the TNM stage. It was observed that the addition of the 5-lncRNA signature led to a 3.6% and 10.7% increase in accuracy in predicting the 2-year and 5-year OS (AUROC 0.668 vs. 0.632 and 0.714 vs. 0.607, respectively; Figure 4). Similar results were obtained by predicting the RFS. When the 5-lncRNA signature was added to the multivariate logistic model, the accuracy levels increased by ~5.5% and 3.7%, respectively, during the prediction of disease-free survival for 2 and 5 years, respectively. These results indicate that the 5-lncRNA signature may have promising survival prediction capabilities in patients with bladder cancer.

### 3.4. lncRNA Signature-Associated Signaling Pathways

**Figure 5:**
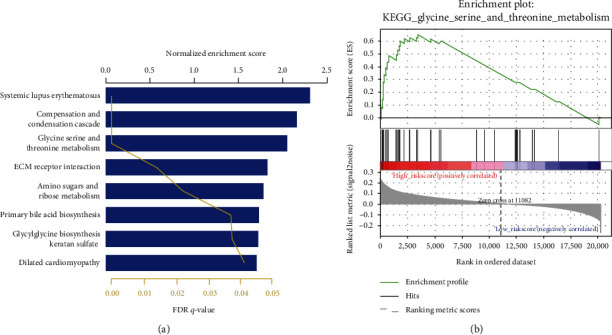
Signature-related lncRNA may affect the biological behavior of bladder cancer through metabolic pathways.

The present study performed gene set enrichment analysis (GSEA) (http://software.broadinstitute.org/gsea/index.jsp) to identify potential relevant signaling pathways and biological processes in MIBC. As illustrated in Figure 5, certain cancer-associated pathways such as systemic lupus erythematosus, glycine serine and threonine metabolism, and glycylglycine biosynthesis of keratin sulfate are enriched in the high-risk patient group, suggesting that the 5-lncRNA signature may be involved in cancer metabolism.

## 4. Discussion

Urothelial carcinoma is the most common type of bladder cancer [16]. Certain novel drugs, such as PD-L1 inhibitors, propose novel therapeutic options and an improved prognosis [17]. However, the heterogeneity of tumors necessitates the exploration of individualized treatments and prognostic biomarkers. Noncoding RNAs have been the focus of a plethora of studies; however, the oncological value of lncRNAs, as a novel type of noncoding RNA, is currently unclear in the clinical setting. Consequently, the present study focused on the clinical application of lncRNAs in bladder cancer and explored the underlying complex biological function involved in various cancer types, including MIBC, such as cell migration and invasion [18]. In addition, certain lncRNAs including UCA1 [19], lncRNA-n336928 [20], HNF1A-AS1 [21], and ZEB2-AS1 [22] were associated with higher grade, higher TNM staging, progression, metastasis, or unfavorable survival results. It has been established previously that the expression of lncRNAs is relatively low, and using a single lncRNA alone may easily introduce bias into the results obtained. The combination of candidate lncRNAs not only improves the accuracy of results but also reduces this difference. In recent years, signatures based on RNA sequencing analysis [23–26] have been developed to identify subgroups that have more aggressive phenotypes or poor survival outcomes in several types of cancer, such as liver cancer [27].

However, the majorities of constructed signatures have failed to describe clinical features and are unable to work in tandem with the clinical needs of patients. The selection of an lncRNA signature was detected between the tumor tissue and adjacent tissues of study participants, which did not accurately reflect the clinical features observed. The present study, to the best of our knowledge, is the first to demonstrate that some nontobacco smokers and smokers who smoked for a shorter period of time exhibited recurrence or metastasis. Conversely, patients who had smoked for a longer period had a good prognosis. Between these two groups, a total of 44 differential expressed lncRNAs were identified. Among these lncRNAs and through excavating the 246 RNA sequencing data from patients with MIBC, a 5-lncRNA signature associated with the AJCC T stage and worse patient outcome was identified. Further multivariate analysis demonstrated that the 5-lncRNA signature was an independent predictor of RFS and OS in patients with MIBC.

To examine the independence of our the 5-lncRNA signature in predicting OS, a multivariate Cox regression analysis was conducted, including age at the time of diagnosis, AJCC T stage, AJCC N stage, lymphovascular invasion, tumor subtype, and pathological stage as covariates. Patients with MIBC with a high AJCC T stage are associated with MIBC-related poor survival prognosis. Similarly, other parameters of malignancy including the AJCC T stage and lymph node status also have a prognostic impact on patient survival. In the univariate analysis, all the examined covariates revealed significant relevance with OS. However, the risk score for 5-lncRNA signature maintains its prognostic impact on OS. Therefore, the present study can conclude that the 5-lncRNA signature identified may serve as an independent prognostic factor for OS. In addition, a positive correlation between the risk score and AJCC T stage was identified. As illustrated in Figure 3, the patients with a higher AJCC T stage typically exhibited higher risk scores (*P* = 0.01). Subgroup analysis based on the AJCC T stage was subsequently performed. Surprisingly, the statistical significance of the survival curve between patients with both the AJCC T stage equal to 2 and AJCC T stages higher than 2 indicates the suitability of this signature for low and high AJCC T stages.

Furthermore, ROC analysis demonstrated that the 5-lncRNA-signature AUROC was equal to 0.668 in 2-year OS predictions, 0.714 in 5-year OS predictions, 0.647 in predicted 2-year RFS, and 0.610 in 5-year RFS predictions. This information exceeds the level of detail previously provided by pathological stages. At present, pathological staging is an element that contributes to risk scores and has been demonstrated to correlate with patient survival [28]. Based on standard treatment, high-risk score patients may require more aggressive treatment or additional adjuvant therapy.

Regarding the characteristics of the five lncRNAs, overexpression of AC090587.5 was correlated with a lower survival rate of overall survivors, while the other four lncRNAs (RP5-827C21.4, ENSG00000182109.3, ENSG00000269609.1, and CTD-2576F9.2) were significantly higher in the high-risk group when compared with the low-risk group. However, the functions of these four lncRNAs in MIBC have not yet been reported. At present, a number of studies have demonstrated that the proliferation of MIBC cell lines may be regulated by specific lncRNAs [29, 30]. For example, upregulated lncRNAs in MIBC tissues was demonstrated to affect the proliferation of MIBC cell lines through the regulation of ZIC2 and the PI3K/AKT signaling pathway [31]. Additionally, the lncRNA XIST that interacted with miR-124 may largely affect the growth, invasion, and migration of MIBC cells [32]. In order to obtain an improved understanding of these lncRNAs in MIBC, additional functional studies should be conducted.

Several limitations of the present study should be considered. Firstly, partial lncRNAs we include were not annotated in the dataset but were still included in the present study. Secondly, the primers of these lncRNAs were not designed; therefore, the signatures in the Fudan University Cancer Center (Shanghai, China) cohort could not be verified. Furthermore, there is a lack of an appropriate mechanism to investigate the prognostic value of lncRNAs in MIBC. In addition, the specific roles of 5 lncRNAs on MIBC phenotypes are unclear. Finally, these findings were summarized in a published dataset without a prospective testing in clinical trials; although the large sample size provides support for these findings, in vitro and in vivo studies should also be performed.

## 5. Conclusion

It is well known that prognosis monitoring is a key issue through the treatment process of MIBC [33]. In the present study, five lncRNA signatures, which correlated with the AJCC N stage, lymph vascular invasion, tumor subtype, and pathological stage, were demonstrated to be independent predictors of OS and RFS. Although not significantly different, the predictive worth of the 5-lncRNA signature was more accurate than that of the pathological stage. Clinically, the 5-lncRNA signature has the potential to be developed into an easy-to-use prognostic model to facilitate further stratification in patients. In addition, the 5-lncRNA signature may have clinical value as a therapeutic target; therefore, the clinical significance and mechanism of action of these five lncRNAs require further study.

## Figures and Tables

**Table 1:** Clinicopathological characteristics of the patients with muscle-invasive bladder cancer.

Characteristics	TCGA cohort (*N* = 246)	
	*N*	%
Age, median (range)	61.00	28–88
Gender		
Male	188	76.4
Female	60	23.6
pT stage		
T2	71	28.9
T3	118	48.0
T4	39	15.9
Tx	18	7.2
pN stage		
N0	149	60.6
N1	26	10.6
N2	46	18.7
N3	6	2.4
Nx	19	7.7
Pathological stage		
Stage II	78	31.7
Stage III	89	36.2
Stage IV	79	32.1
Histological grade		
Low grade	19	7.7
High grade	227	92.3
Family history of cancer		
Yes	83	33.7
No	163	66.3
Smoking status		
Lifelong nonsmoker	61	24.8
Current smoker	55	22.4
Current reformed smoker for >15 years	66	26.8
Current reformed smoker for ≤15 years	46	18.7

**Table 2:** The lncRNA after limma package filter for FC > 1.5 and *P* < 0.05.

ID	LogFC	AveExpr	*t*	*P* value	Adj. *P* value	*B*
Group	1	0.5	1700.905	1.16*E*-63	1.47*E*-59	99.32529
ENSG00000214049.6	-66.98181715	36.94566334	-2.14017	0.042493	0.86393	-6.6734
ENSG00000267308.1	-44.63341851	24.65613284	-2.13682	0.042791	0.86393	-6.67968
ENSG00000271738.1	-3.767529991	5.066329131	-2.39732	0.024477	0.86393	-6.17191
ENSG00000247556.2	-2.135716344	5.685292246	-2.57665	0.016407	0.86393	-5.80145
ENSG00000272003.1	-2.005958624	3.639689341	-2.35069	0.027107	0.86393	-6.26557
ENSG00000236028.1	-1.374460766	0.745926543	-2.5264	0.018375	0.86393	-5.90686
ENSG00000204792.2	-0.831347221	0.903531328	-3.28434	0.003073	0.86393	-4.20749
ENSG00000272933.1	-0.60593197	0.908743886	-2.09887	0.046305	0.86393	-6.75036
ENSG00000259244.1	-0.585575194	0.852920315	-2.40473	0.024082	0.86393	-6.15693
ENSG00000228526.2	-0.58555543	0.731767099	-2.07405	0.048742	0.86393	-6.79613
ENSG00000228661.1	-0.58523181	1.016775755	-2.19205	0.038103	0.86393	-6.57525
ENSG00000182109.3	0.585048988	0.543219496	2.520216	0.018632	0.86393	-5.91974
ENSG00000236305.1	0.585276951	0.69807265	2.145152	0.042052	0.86393	-6.66403
ENSG00000269609.1	0.597696649	1.394042361	2.085292	0.047625	0.86393	-6.77544
ENSG00000257925.1	0.654059885	0.575967518	2.074136	0.048733	0.86393	-6.79596
ENSG00000272667.1	0.680461666	1.299456088	2.181024	0.039	0.86393	-6.59624
ENSG00000273066.1	0.702976896	0.806816281	2.230055	0.035152	0.86393	-6.50235
ENSG00000186019.10	0.730220195	0.629624554	2.50134	0.019436	0.86393	-5.95897
ENSG00000270017.1	0.741773525	1.281822856	2.131753	0.043246	0.86393	-6.68916
ENSG00000233368.2	0.744301473	1.709706841	2.144091	0.042145	0.86393	-6.66603
ENSG00000246174.3	0.756055369	0.825006424	2.175208	0.039481	0.86393	-6.60728
ENSG00000229950.1	0.798871137	1.384972482	2.064272	0.049732	0.86393	-6.81404
ENSG00000272455.1	0.810152095	1.68041124	2.252562	0.033503	0.86393	-6.45879
ENSG00000235919.3	1.122413784	2.001191101	2.078657	0.048281	0.86393	-6.78765
ENSG00000229967.1	1.139925487	0.652604965	2.496279	0.019657	0.86393	-5.96945
ENSG00000228714.2	1.160413191	0.825818632	2.346047	0.027382	0.86393	-6.27483
ENSG00000233527.4	1.201871884	1.607995948	2.293887	0.030658	0.86393	-6.37807
ENSG00000266602.1	1.22544269	0.751986675	2.675669	0.013092	0.86393	-5.59031
ENSG00000231663.1	1.296743096	1.976506657	2.088598	0.0473	0.86393	-6.76934
ENSG00000197989.9	1.508941473	4.522039745	2.322033	0.028849	0.86393	-6.32254
ENSG00000273281.1	1.511092559	2.581216972	2.692029	0.012608	0.86393	-5.555
ENSG00000270372.1	1.740174215	1.169668163	2.266858	0.032493	0.86393	-6.43097
ENSG00000170846.11	2.105970884	5.75109779	2.787283	0.010112	0.86393	-5.34714
ENSG00000272960.1	2.36600947	3.319955745	4.559335	0.000122	0.775456	-1.02704
ENSG00000184324.11	2.664136784	1.586197272	2.520825	0.018606	0.86393	-5.91847
ENSG00000232956.4	2.762300952	5.413196686	3.025176	0.005762	0.86393	-4.81231
ENSG00000196756.7	3.108853067	6.656784779	2.081814	0.047968	0.86393	-6.78185
ENSG00000175701.6	3.367781939	7.184080975	2.219151	0.035977	0.86393	-6.52335
ENSG00000204588.5	3.972279381	6.155563889	2.243726	0.034142	0.86393	-6.47593
ENSG00000255135.3	4.0897323	5.960712655	2.444255	0.02207	0.86393	-6.07651
ENSG00000197463.8	4.244524784	2.889874234	2.604638	0.015398	0.86393	-5.74222
ENSG00000254024.1	9.057860809	7.223835279	2.126317	0.043739	0.86393	-6.69932
ENSG00000234741.3	12.24952015	24.77846878	2.371656	0.025894	0.86393	-6.2236
ENSG00000260260.1	19.48162502	27.22555856	2.305031	0.02993	0.86393	-6.35613

**Table 3:** Correlations between the risk score of the 5-lncRNA signature and clinicopathological characteristics.

Variable	Low-risk group (*n* (%) = 123)	High-risk group (*n* (%) = 123)	Pearson *X*^2^	*P* value
Age of diagnosis			1.598	0.450
≤65	48	46		
>65	75	77		
AJCC T stage			**9.315**	**0.009**
T2	45	25		
T3+T4	67	88		
Lymphonodus status			**4.019**	**0.045**
Negative	82	67		
Positive	32	46		
Tumor subtype			4.423	0.110
Nonpapillary	76	88		
Papillary	46	31		
Pathological stage			**4.058**	0.105
Stage II+stageIII	91	76		
Stage IV	32	47		
Histological grade			**16.787**	**<0.001**
Low grade	18	1		
High grade	105	122		
Family history			0.641	0.726
Yes	40	43		
No	83	80		
Smoking status^a^			1.112	
Nonsmoker and smoker for ≤15 years	53	54		
Smoker for >15 years	36	30		

Statistical significant results (in bold).

**Table 4:** Univariate and multivariate Cox regression analyses of five-lncRNA signature in prediction of overall survival.

Variable	Univariate analysis			Multivariate analysis		
	HR	95% CI	*P* value	HR	95% CI	*P* value
Risk score (high vs. low)	2.720	1.659–4.460	**<0.001**	2.210	0.845–2.430	**<0.001**
Age	1.027	1.008–1.047	**<0.006**	1.032	1.009–1.054	**0.005**
Gender	0.971	0.622–1.514	0.895			
Tumor subtype	1.916	1.182–3.105	**0.008**	1.503	0.872–2.591	0.208
T stage (T2 vs. T3-4)	1.996	1.227–3.247	**0.005**	1.433	0.845–2.430	0.182
N starge^a^	2.185	1.461–3.270	**<0.001**	1.848	1.212–2.819	**0.004**
Pathological stage (II-III vs. IV)	2.051	1.393–3.201	**<0.001**	1.848	1.212–2.819	**0.004** ^**b**^
Histological grade	3.130	0.770–12.723	0.111			
Family history	0.868	0.579–1.301	0.493			
Smoking status^a^	1.428	0.903–2.258	0.128			

^a^Binary variables: no lymph node involvement versus otherwise; nonmetastasis versus otherwise; nonsmoker and smoker for ≤15 years versus smoker for >15 years. ^b^Constant or linear covariance makes N stage = pathological stage. HR: hazard ratio; CI: confidential interval; vs.: versus. Statistically significant results (in bold).

**Table 5:** Univariate and multivariate Cox regression analyses of the 5-lncRNA signature in the prediction of RSF.

Variable	Univariate analysis			Multivariate analysis		
	HR	95% CI	*P* value	HR	95% CI	*P* value
Risk score (high vs. low)	3.008	1.652–5.476	**<0.001**	2.637	1.428–4.869	**0.002**
Age	1.098	0.659–1.831	0.720	1.386	0.761–2.524	0.286
Gender	1.051	0.588–1.881	0.867			
Tumor subtype	1.916	1.182–3.105	**0.016**			
T stage (T2 vs. T3-4)	2.627	1.319–5.234	**0.006**	2.024	0.923–4.436	0.078
N stage^a^	2.346	1.380–3.990	**<0.002**	1.914	1.085–3.378	**0.025**
Pathological stage (II-III vs. IV)	2.051	2.233–3.695	**<0.002**	1.914	1.085–3.378	**0.025** ^b^
Histological grade	5.396	0.743–38.816	0.096			
Family history	1.136	0.684–1.886	0.622			
Smoking status^a^	0.805	0.453–1.432	0.460			

^a^Binary variables: no lymph node involvement versus otherwise; nonsmoker and smoker for ≤15 years versus smoker for >15 years. ^b^Constant or linear covariance makes N stage = pathological stage. HR: hazard ratio; CI: confidential interval; vs.: versus. Statistical significant results (in bold).

## Data Availability

The data used to support this study can be available from http://xena.ucsc.edu/ and http://bioinformatics.mdanderson.org/main/TANRIC:Overview.

## Supplementary Materials

Supplementary Materials Supplementary Figure 1: Kaplan-Meier estimates of RSF of patients with MIBC between high-risk and low-risk groups using the 5-lncRNA signature. RSF: relapse-free survival. Supplementary Table 1: the group after the propensity score matching.

## References

[1] Society AC (2015). *Cancer facts and figures 2015*.

[2] Chen W., Zheng R., Baade P. D. (2016). Cancer statistics in China, 2015. *CA: a Cancer Journal for Clinicians*.

[3] Kessler T. M., Burkhard F. C., Studer U. E. (2005). Clinical indications and outcomes with nerve-sparing cystectomy in patients with bladder cancer. *The Urologic Clinics of North America*.

[4] Kaasinen E., Rintala E., Hellström P. (2002). Factors explaining recurrence in patients undergoing chemoimmunotherapy regimens for frequently recurring superficial bladder carcinoma. *European Urology*.

[5] Miladi M., Peyromaure M., Zerbib M., Saighi D., Debre B. (2003). The value of a second transurethral resection in evaluating patients with bladder tumours. *European Urology*.

[6] Larsson P., Wijkstrom H., Thorstenson A. (2003). A population-based study of 538 patients with newly detected urinary bladder neoplasms followed during 5 years. *Scandinavian Journal of Urology and Nephrology*.

[7] Davarpanah N. N., Yuno A., Trepel J. B., Apolo A. B. (2017). Immunotherapy: a new treatment paradigm in bladder cancer. *Current Opinion in Oncology*.

[8] Steiner H., Bergmeister M., Verdorfer I. (2008). Early results of bladder-cancer screening in a high-risk population of heavy smokers. *BJU International*.

[9] Yu D., Geng H., Liu Z. (2017). Cigarette smoke induced urocystic epithelial mesenchymal transition via MAPK pathways. *Oncotarget*.

[10] Chen L. M., Nergard J. C., Ni L., Rosser C. J., Chai K. X. (2013). Long-term exposure to cigarette smoke extract induces hypomethylation at the RUNX3 and IGF2-H19 loci in immortalized human urothelial cells. *PLoS One*.

[11] Ogihara K., Kikuchi E., Yuge K. (2016). Refraining from smoking for 15 years or more reduced the risk of tumor recurrence in non-muscle invasive bladder cancer patients. *Annals of Surgical Oncology*.

[12] Zhu Z., Xu L., Wan Y. (2018). Inhibition of E-cadherin expression by lnc-RNA H19 to facilitate bladder cancer metastasis. *Cancer Biomarkers*.

[13] Li J., Han L., Roebuck P. (2015). TANRIC: an interactive open platform to explore the function of lncRNAs in cancer. *Cancer Research*.

[14] Ritchie M. E., Phipson B., Di Wu Y. H., Law C. W., Shi W., Smyth G. K. (2015). limma powers differential expression analyses for RNA-sequencing and microarray studies. *Nucleic Acids Research*.

[15] Huang T.-b., Dong C.-p., Zhou G.-c. (2017). A potential panel of four-long noncoding RNA signature in prostate cancer predicts biochemical recurrence-free survival and disease-free survival. *International Urology and Nephrology*.

[16] Grivas P. D., Melas M., Papavassiliou A. G. (2015). The biological complexity of urothelial carcinoma: insights into carcinogenesis, targets and biomarkers of response to therapeutic approaches. *Seminars in Cancer Biology*.

[17] Aoun F., Kourie H. R., Sideris S., Roumeguère T., van Velthoven R., Gil T. (2015). Checkpoint inhibitors in bladder and renal cancers: results and perspectives. *Immunotherapy*.

[18] Xue M., Pang H., Li X., Li H., Pan J., Chen W. (2016). Long non-coding RNA urothelial cancer-associated 1 promotes bladder cancer cell migration and invasion by way of the hsa-miR-145-ZEB1/2-FSCN1 pathway. *Cancer Science*.

[19] Li Z., Li X., Wu S., Xue M., Chen W. (2014). Long non-coding RNA UCA1 promotes glycolysis by upregulating hexokinase 2 through the mTOR-STAT3/microRNA143 pathway. *Cancer Science*.

[20] Chen T., Xie W., Xie L. (2015). Expression of long noncoding RNA lncRNA-n336928 is correlated with tumor stage and grade and overall survival in bladder cancer. *Biochemical and Biophysical Research Communications*.

[21] Zhan Y., Li Y., Guan B. (2017). Long non-coding RNA HNF1A-AS1 promotes proliferation and suppresses apoptosis of bladder cancer cells through upregulating Bcl-2. *Oncotarget*.

[22] Wu X., Yan T., Wang Z., Wu X., Cao G., Zhang C. (2017). LncRNA ZEB2-AS1 promotes bladder cancer cell proliferation and inhibits apoptosis by regulating miR-27b. *Biomedicine & Pharmacotherapy*.

[23] Meng J., Li P., Zhang Q., Yang Z., Fu S. (2014). A four-long non-coding RNA signature in predicting breast cancer survival. *Journal of Experimental & Clinical Cancer Research*.

[24] Zhang X.-Q., Sun S., Lam K.-F. (2013). A long non-coding RNA signature in glioblastoma multiforme predicts survival. *Neurobiology of Disease*.

[25] Zhou M., Zhong L., Xu W. (2016). Discovery of potential prognostic long non-coding RNA biomarkers for predicting the risk of tumor recurrence of breast cancer patients. *Scientific Reports*.

[26] Shukla S., Evans J. R., Malik R. (2016). Development of a RNA-Seq based prognostic signature in lung adenocarcinoma. *Journal of the National Cancer Institute*.

[27] Zhang J., Li Z., Liu L. (2018). Long noncoding RNA TSLNC8 is a tumor suppressor that inactivates the interleukin-6/STAT3 signaling pathway. *Hepatology (Baltimore, Md)*.

[28] Hansel D. E., Amin M. B., Comperat E. (2013). A contemporary update on pathology standards for bladder cancer: transurethral resection and radical cystectomy specimens. *European Urology*.

[29] Luo M., Li Z., Wang W., Zeng Y., Liu Z., Qiu J. (2013). Long non-coding RNA H19 increases bladder cancer metastasis by associating with EZH2 and inhibiting E-cadherin expression. *Cancer Letters*.

[30] Zhong X., Long Z., Wu S., Xiao M., Hu W. (2018). LncRNA-SNHG7 regulates proliferation, apoptosis and invasion of bladder cancer cells assurance guidelines. *Journal of Balkan Union of Oncology*.

[31] Wang J., Ma W., Liu Y. (2017). Long non-coding RNA HULC promotes bladder cancer cells proliferation but inhibits apoptosis via regulation of ZIC2 and PI3K/AKT signaling pathway. *Cancer Biomarkers*.

[32] Xiong Y., Wang L., Li Y., Chen M., He W., Qi L. (2017). The long non-coding RNA XIST interacted with miR-124 to modulate bladder cancer growth, invasion and migration by targeting androgen receptor (AR). *Cellular Physiology and Biochemistry : International Journal of Experimental Cellular Physiology, Biochemistry, and Pharmacology*.

[33] Kamat A. M., Hahn N. M., Efstathiou J. A. (2016). Bladder cancer. *The Lancet*.

