# Supplementary material for: A 5-lncRNA Signature Associated with Smoking Predicts the Overall Survival of Patients with Muscle-Invasive Bladder Cancer
Source: Dis Markers. 2021 Jan 18;2021:8839747. doi: 10.1155/2021/8839747 (PMC7914096; doi:10.1155/2021/8839747)
Supplement: Supplementary Materials — Supplementary Figure 1: Kaplan-Meier estimates of RSF of patients with MIBC between high-risk and low-risk groups using the 5-lncRNA signature. RSF: relapse-free survival. Supplementary Table 1: the group after the propensity score matching. [file 8839747.f1.zip › Supplementary Descrption.doc]

**Supplementary**

**Supplementary Figure 1.** Kaplan–Meier estimates of RSF of patients with MIBC between high-risk and low-risk using the five-lncRNA signature. RSF=Relapse-free survial

**Supplementary Table 1:** The group after the Propensity Score Matching
